# The Role of E3 Ubiquitin Ligases in Chloroplast Function

**DOI:** 10.3390/ijms23179613

**Published:** 2022-08-25

**Authors:** Katherine A. Hand, Nitzan Shabek

**Affiliations:** Department of Plant Biology, College of Biological Sciences, University of California, Davis, CA 95616, USA

**Keywords:** ubiquitin, E3 ligase, chloroplast, stress, photosynthesis, homeostasis, SP1, COP1, PUB4, CHIP, TT3.1, CDC48, protein degradation

## Abstract

Chloroplasts are ancient organelles responsible for photosynthesis and various biosynthetic functions essential to most life on Earth. Many of these functions require tightly controlled regulatory processes to maintain homeostasis at the protein level. One such regulatory mechanism is the ubiquitin-proteasome system whose fundamental role is increasingly emerging in chloroplasts. In particular, the role of E3 ubiquitin ligases as determinants in the ubiquitination and degradation of specific intra-chloroplast proteins. Here, we highlight recent advances in understanding the roles of plant E3 ubiquitin ligases SP1, COP1, PUB4, CHIP, and TT3.1 as well as the ubiquitin-dependent segregase CDC48 in chloroplast function.

## 1. Introduction

As essential and ancient organelles originating from the endosymbiosis of a cyanobacterial organism, chloroplasts are a defining feature of plants and play crucial roles during growth and development [1,2,3]. They are bound by a double membrane envelope surrounding a stromal matrix containing a unique thylakoid membrane home to photosynthesis, a process of transforming CO_2_ into carbohydrates [2,4]. Chloroplasts also perform an array of important biosynthetic processes such as nitrate assimilation and the synthesis of fatty acids, amino acids, and terpenes [5,6]. To maintain these functions, the chloroplast’s diverse proteome must be dynamically controlled.

The chloroplast proteome is known to contain approximately 3000 proteins. More than 95% of the proteome is nuclear-encoded and the remaining ~100 proteins are encoded by the chloroplast genome [5,7,8]. Import of nuclear-encoded chloroplast proteins relies on translocation through translocons known as TOC (translocon at the outer envelope membrane of chloroplasts) and TIC (translocon at the inner envelope membrane of chloroplasts) [9,10,11]. Regulation of the content and quality of the chloroplast proteome is established by several homeostatic mechanisms, including proteolysis, transcriptional control, and translation [9,12]. The presence of damaged or incorrectly sorted proteins can be detrimental and lead to the formation of aggregates or malfunction of important cellular pathways [9,13,14,15]. For instance, several abiotic stresses (i.e., extreme temperature, strong light, and oxidative conditions) can damage properly folded and sorted chloroplast proteins, consequently compromising the integrity of the chloroplast proteome and affecting plant growth [16,17]. Thus, it is imperative that chloroplasts maintain proteome homeostasis.

Recent findings demonstrate a link between the ubiquitin-proteasome system (UPS) and chloroplast function. Notably, plants heavily utilize the UPS to rapidly respond to the ever-changing environment [18,19,20,21,22]. The destruction of a protein by the UPS involves two successive steps: first, conjugation of the protein substrate through covalent attachment of ubiquitin molecules in a process called ubiquitination, and second, degradation of the ubiquitin-conjugated substrate by the 26S proteasome [23,24]. The mechanism of ubiquitination involves the collaborative action of three main enzymes. First, a ubiquitin-activating enzyme (E1) activates ubiquitin through ATP hydrolysis and delivers the activated ubiquitin to a ubiquitin-conjugating enzyme (E2), forming a thioester bond in its cysteine active site with the C-terminus glycine of the activated ubiquitin. Thereafter, a ubiquitin ligase (E3) strictly mediates the transfer of the activated ubiquitin from E2 to the target substrate [13,25]. The last step in the ubiquitination process requires a successive addition of ubiquitin moieties to the Lys residue of the previously conjugated ubiquitin molecule, such that a polyubiquitin chain is formed. Ubiquitin has seven internal lysine residues (Lys6, 11, 27, 29, 33, 48 and 63) that all can potentially be used for polyubiquitin chain formation [26]. The isopeptide linkage within a polyubiquitin chain plays role in determining the cellular fate and function of the modified target protein. In a typical scenario, polyubiquitin chains are linked through Lys48 and target the ubiquitinated proteins for degradation by the 26S proteasome [13,27,28,29].

Remarkably, the Arabidopsis genome contains thousands of proteins (~6% of the total genome) involved in the ubiquitin system, approximately 90% of which are E3 ubiquitin ligases whose functions are highly dynamic and tightly controlled [1,13,28,30]. Given their differences in structure and function, E3 ubiquitin ligases are generally classified into 4 groups: RING (Really Interesting New Gene), HECT (Homologous to the E6AP Carboxyl Terminus), PUB (Plant U-box), and RBR (RING-in-Between-RING) [13,28,31,32]. Within the last decade, advances have been made in illuminating how the UPS and particularly E3 ubiquitin ligases regulate chloroplast function. In this review, we focus on recent progress in understanding the role of E3 ubiquitin ligases in the degradation of chloroplast proteins, which is required for the proper maintenance of the chloroplast proteome.

## 2. Regulation of Chloroplast Function by E3 Ubiquitin Ligases

### 2.1. SP1, a Chloroplast-Localized RING-Type E3 Ligase

To maintain proper chloroplast function, >95% of chloroplast proteins are nuclear-encoded and rely on chloroplast import machinery to enter the organelle [5,7,8]. This import machinery is comprised of TOC and TIC protein complexes [9,10,11]. TOC complexes participate in both protein recognition and import pathways, and as such, can exist in various isoforms to mediate substrate specificity [4,33,34,35]. Studies have demonstrated the role of an E3 ubiquitin ligase, suppressor of plastid protein import 1 (*ppi1)* locus 1 (SP1), in alternating between different compositions of TOC isoforms to fine tune substrate-specific import [36].

**Figure 1:**
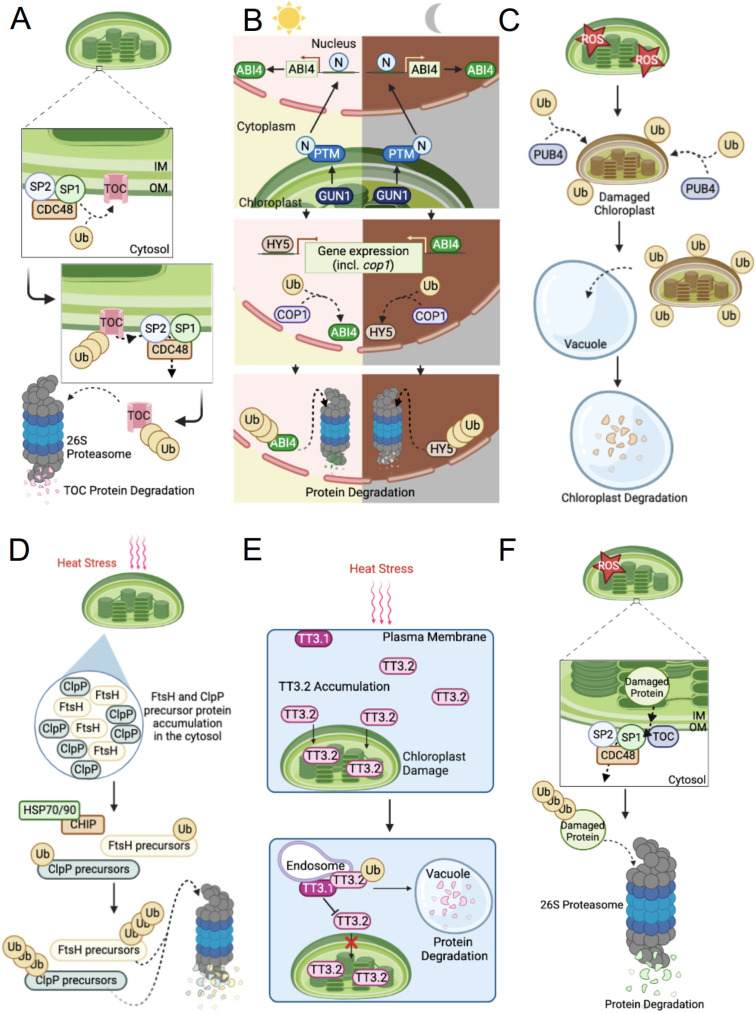
Schematic representation of the mechanisms of E3 ubiquitin ligases in regulating chloroplast function. (**A**) SP1-mediated degradation of TOC machinery through the CHLORAD pathway. The specific subunits targeted (Toc33/34/75/120/132 and Toc159) are represented as TOC (detailed in Section 2.1). (**B**) Light and dark pathways of the feedback mechanism involving COP1, HY5, and ABI4 in chloroplast development (detailed in Section 2.2). (**C**) Role of PUB4 in chloroplast degradation (detailed in Section 2.3). (**D**) CHIP-mediated degradation of chloroplast protease precursor proteins in the cytosol (detailed in Section 2.4). (**E**) Simplified schematic of TT3.1-mediated degradation of TT3.2 under heat stress (detailed in Section 2.5). (**F**) CHLORAD pathway involving SP1 and CDC48 (detailed in Section 3). Ub, ubiquitin; OM, outer membrane; IM, inner membrane; PTM, plant homeodomain type transcription factor with transmembrane domains. All images were created with BioRender.com.

SP1, a chloroplast-localized RING-type E3 ligase and outer membrane protein (Omp), was first identified in a mutagenesis screen investigating suppressors of *ppi1*, a Toc33 mutant shown to reduce chloroplast protein import and produce chlorosis [4,37]. The screen displayed defects in Toc33 and Toc75 genes impacting the proper function of protein import and plant growth [37]. Additional SP1 overexpression and mutant analysis confirmed its role in reconfiguring TOC machinery by ubiquitination and subsequent degradation by the 26S proteasome [4,37]. For instance, decreased accumulation of photosynthetic proteins and disproportionate TOC receptor levels were observed in *sp1* single mutants which led to poor photosynthetic performances affecting leaf senescence and de-etiolation [37]. In the same study, SP1 was revealed to interact with and ubiquitinate several TOC proteins, including the *Arabidopsis thaliana* (At) AtToc159, AtToc132, AtToc120, AtToc75, AtToc34, and AtToc33 (Figure 1A) [37]. Recent studies propose additional roles for SP1 in chloroplast development and protein import involving plastid interconversion events and tolerance to various stresses (i.e., salt, osmosis, abiotic, and/or ROS production) [37,38].

Furthermore, a chloroplast-associated protein degradation pathway (CHLORAD) was shown to target damaged TOC machinery [7]. In this pathway, SP1 interacts with Omp85-type β-barrel channel protein suppressor of *ppi1* locus2 (SP2) and cell division control protein 48 (CDC48), a conserved AAA+ (ATPases associated with diverse cellular activities) chaperone, to form a complex that mediates TOC protein degradation via protein retrotranslocation and the UPS (Figure 1A) [7,39]. While SP1 is involved in mediating the process of ubiquitination in the presence of stress or developmental cues, the exact mode of target substrate recognition by SP1 remains largely elusive [7,37]. Interestingly, it has been shown that SP1 can be auto-ubiquitinated, leading to its self-degradation by the 26S proteasome through CHLORAD [7]. However, the processes regulating SP1 and the existence of other chloroplast-localized E3 ligases remain to be revealed [36].

Notably, a crucial role for SP1 and the CHLORAD pathway has been discovered in the regulation of fruit ripening in tomato plants [33]. In view of this, two evolutionarily conserved chloroplast SP1 homologues have been studied: SPL1 and SPL2, whose roles are still largely unknown [37,40]. Knockdown expression analysis of SP1 and SPL2 demonstrated delays in leaf senescence and fruit ripening while the opposite effect was apparent in the overexpression analysis of SP1. In addition, SP1 knockdown and RNA analyses on tomato fruit development exhibited changes in color, firmness, and ripening-related gene levels [33]. This indicated that SP1 indirectly induces the retrograde signals required for ripening, influences gene expression, and directly reconfigures TOC complexes to promote the import of ripening-related proteins (i.e., ethylene synthesis, carotenoid synthesis, cell wall modification, lipid metabolism, and chlorophyll catabolism proteins).

### 2.2. RING-Type E3 Ligase, COP1

Light is essential for all life and serves as an important signal not only for plant flowering but also the expression of genes required to construct photosynthetically competent chloroplasts [6,41,42,43]. In view of this, chloroplast populations are reliant upon light signals in regulating multiple phytohormones, such as ethylene, brassinosteroid, cytokinin, abscisic acid (ABA), auxin, and gibberellin, which play fundamental roles in modulating photosynthetic processes and chloroplast development at the transcriptional level [44,45,46]. These phytohormone pathways are directly or indirectly controlled by the UPS, and specifically some ubiquitin ligases participate in hormone perception and signaling mechanisms [22,47]. Here, we highlight yet another role for the UPS-phytohormones crosstalk in chloroplast function and development.

Importantly, the construction of photosynthetically competent chloroplasts also requires the import of nuclear-encoded precursor proteins, chlorophyll biosynthesis, and formation of the thylakoid membranes [6,48]. These activities rely on retrograde signaling to manage the expression of photosynthesis-associated nuclear genes (*PhANG*s) [49,50,51,52]. One essential process controlled by the combination of light and phytohormone pathways is seedling de-etiolation, a process of developing functional chloroplasts during the transition from heterotrophic to photoautotrophic growth in plants [42,53,54,55]. To maintain properly scheduled seedling de-etiolation and photomorphogenesis, Constitutive Photomorphogenesis 1 (COP1), a RING-type E3 ligase, regulates these processes during the dark-to-light shift sensed by photoreceptors (i.e., phytochromes and cryptochromes) [56,57,58,59]. The activation of COP1 occurs in the dark when it accumulates in the nucleus and a complex with Suppressor of PhyA-105 (SPA) is formed to polyubiquitinate activated photoreceptors and positive regulators of light signaling [58,60,61,62,63]. Conversely, COP1-mediated degradation of these positive regulators is inhibited upon light exposure [56,59,60]. In addition, the coordinated efforts involved in chloroplast development also rely on the repressive abilities of Phytochrome Interacting Factors (PIFs) (i.e., PIF1 and PIF3) and Ethylene-Insensitive 3 (EIN3) in repressing genes associated with chlorophyll biosynthesis and etiolation [64,65,66].

Recently, several studies have illuminated an intriguing connection linking hormone, light, and retrograde signaling pathways to the fundamental role of COP1-mediated degradation in chloroplast biogenesis [44,53,67]. ABA is a plant hormone participating in stress signaling and functions antagonistic to light signaling to control developmental processes such as seed germination and stomatal movement [68,69,70]. A feedback mechanism regulating transcription factors ABA Insensitive 4 (ABI4) and Elongated Hypocotyl 5 (HY5), involved in mediating retrograde signaling/plant development and promoting de-etiolation, respectively, revealed that COP1 serves as a convergence point for the integration of light and chloroplast signaling pathways [53,71]. Here, a chloroplast signal mediated by GUN1 and the N-terminus of chloroplast envelope-bound plant homeodomain type transcription factor with transmembrane domains (PTM) activates ABI4. Accordingly, ABI4 and HY5 are shown to regulate COP1 expression while COP1 targets ABI4 in the light and HY5 in the dark for proteasomal degradation (Figure 1B). Regulating the functions of ABI4, HY5, and COP1 ensures the establishment of functional chloroplasts; however, the exact mechanisms underlying chloroplast retrograde signals remain unknown [53]. Similarly, increased COP1 activity was observed under long-term ABA treatments which also displayed elevated levels of Golden2-Like1 (GLK1) degradation [67]. Importantly, the degradation of GLK1, a key transcription factor in regulating *PhANG* expression encoding chlorophyll biosynthesis enzymes, led to varying levels of leaf yellowing indicative of the suppression of chloroplast development in a light-dependent manner under both long-term ABA and different light intensity treatments [67,72].

Furthermore, COP1 also participates in managing several negative regulators in chloroplast biogenesis. For instance, Brassinazole-Resistant 1 (BZR1), a negative regulator of photomorphogenesis and chloroplast development, is indicated as a target of COP1. In the light, active (dephosphorylated) BZR1 is inhibited by HY5 to enable the progression of chloroplast development whereas COP1 targeted inactive forms of BZR1 in the dark for degradation [44,73,74]. Moreover, COP1 has also been shown to interact non-proteolytically with negative regulators of *PhANG*s, such as PIF3 and EIN3. For instance, COP1 prevents the phosphorylation of PIF3 and preys on E3 ubiquitin ligases EIN3-Binding F-box protein 1 and 2 (EBF1 and EBF2) who target EIN3. Thus, stabilizing both PIF3 and EIN3 and inhibiting their subsequent degradation [75]. Interestingly, COP1 engages with cytoplasmic processing bodies (p-bodies), granules that can function to store, degrade, and translate mRNA. *Dcp5-1*, a defective p-body mutant in Arabidopsis, coupled with *cop1-6* mutants demonstrated the need for COP1 in promoting the formation of p-bodies in the dark that attenuate the translation of specific mRNAs for chlorophyll biosynthesis [76]. Taken together, these recent studies have revealed that the role of COP1 extends far beyond its involvement in light signaling. COP1 is a crucial component mediating substrate-specific protein levels as well as light, hormone, and retrograde signaling pathways, all of which are essential in chloroplast development and function.

### 2.3. PUB4, a Cytosolic Plant U-Box E3 Ubiquitin Ligase

Chloroplasts accumulate oxidative damage in the presence of reactive oxygen species (ROS), such as singlet oxygen (^1^O_2_), superoxide anion radicals, and hydrogen peroxide produced during photosynthesis [4,16,77,78]. This can occur when the organelle’s photosynthetic electron transport chain exceeds its capacity to transfer electrons under stressful environmental conditions resulting in electron leakage [78,79,80]. Thus, quality control mechanisms are needed to respond to these injuries and enable chloroplast turnover for the redistribution of nutrients [81,82,83]. To do so, chloroplasts can communicate information about their condition with the nucleus by retrograde signaling pathways to influence gene expression as well as utilize regulatory mechanisms such as the ubiquitin system or autophagy [49,50,51,52,79,81,84].

While investigating the mechanisms underlying these processes, genetic screens investigating plastid ferrochelatase enzyme mutants and ^1^O_2_-induced chloroplast degradation in Arabidopsis revealed Plant U-Box 4 (PUB4), a cytosolic E3 ubiquitin ligase that targets unknown plastid proteins and chloroplasts for vacuolar-dependent degradation [79,85]. Here, Woodson et al. focused on ferrochelatase 1 and 2 (FC1 and FC2) which are conserved plastid enzymes, at the heme-chlorophyll branch point in the tetrapyrrole biosynthetic pathway that function by converting protoporphyrin IX to heme; a conversion involved in retrograde signaling [79,86,87]. In this study, mutant plant lines *fc2-1* and *fc2-2* promoted photosynthetic cell death in the cotyledons and an overproduction of both ^1^O_2_ and a photosensitizing intermediate, Proto, within chloroplasts [79]. Notably, elevated levels of nuclear stress-associated genes, ROS, and damaged chloroplasts were reported in the *fc2-1/pub4-6* double mutants. Most noteworthy was the observation that ROS-damaged chloroplasts were specifically targeted by PUB4 and required a ^1^O_2_-generated signal involving PUB4 (Figure 1C) [79].

Recently, a study analyzed a variety of mutants in conjunction with an Arabidopsis *gun1-102 ftsh5-3* double mutant known to promote the degradation of damaged chloroplasts and the formation of a variegated phenotype in the cotyledons and leaves. Chloroplast-localized Genomes Uncoupled-1 (GUN1) protein mediates chloroplast-to-nucleus signals whereas Filamentous temperature sensitive H5 (FtsH5) protein is involved in chloroplast development and photosystem repair. Importantly, the *gun1-102 ftsh5-3 pub4-7* triple mutant prevented the degradation of damaged chloroplasts and did not produce the variegated phenotype [88]. These findings also demonstrate PUB4 as a key player in chloroplast degradation to maintain plastid protein homeostasis. Nonetheless, its target substrates and the precise mechanisms by which it operates remain unknown [81].

Although various systems of protein degradation may indeed exist in chloroplasts, it remains unclear how the organelle is maintained by different degradation systems simultaneously. An investigation into two major eukaryotic degradation pathways, autophagy and the UPS, analyzed *pub4-6 atg5-1* and *pub4-6 atg7-2* double mutants [89]. Both double mutants displayed higher levels of ROS, nitrogen and carbon starvation susceptibility, early cell death, and lower seed production in comparison to wild-type or single mutants [85,89]. Cleavage assays investigating the activity of chloroplast autophagy displayed similar levels of autophagy activity in both wild-type and *pub4-6* mutants under high light treatment [89]. These findings revealed that PUB4-mediated degradation and autophagy act in parallel to maintain proper control of the proteome [85,89]. Interestingly, several recent studies have further revealed additional roles of PUB4 in plant growth and development, including shoot and root meristem regulation, cytokinin signaling, and microbe-associated molecular pattern-triggered immunity [90,91,92,93]. Given the versatility of its function, future studies may shed light on how exactly PUB4 is able to regulate both plant and chloroplast pathways to maintain homeostasis.

### 2.4. Chaperone-Dependent U-Box Containing E3 Ligase, CHIP

Owing to the large number of nuclear-encoded proteins comprising the chloroplast proteome, coordinated transport following synthesis in the cytosol to the stroma is critical for the proper function of the organelle [5,12,94]. Thereafter, these precursor proteins are subjected to proteolytic processing for the cleavage of their transit peptides and undergo folding, and/or sorting with the assistance of molecular chaperones to other compartments, such as the thylakoids [5,95,96,97]. Importantly, chloroplast precursor proteins must be unfolded for translocation [98,99]. Left unfolded and unregulated, they tend to form non-specific protein aggregates in the cytosol [97,100,101]. To date, plants contain >20 chloroplast proteases, including chloroplast caseinolytic proteases (Clps) in the stroma and FtsHs on the thylakoid membranes which are both involved in the removal of imported and chloroplast-encoded proteins [102,103,104,105,106]. Remarkably, a cytosolic E3 ubiquitin ligase was discovered to regulate these chloroplast proteases.

Carboxyl Terminus of the Hsp70-Interacting Proteins (CHIP), a highly conserved chaperone-dependent and U-box containing cytosolic E3 ligase, targets chloroplast protease precursor proteins to prevent their accumulation and aggregation in the cytosol [96,107,108,109,110,111]. Targeting these substrates requires the help of heat shock protein 70 or 90 (Hsp70 or Hsp90), molecular chaperones who monitor misfolded proteins [96,112]. A study investigating Arabidopsis *ppi2* mutant plants, shown to impair protein import into chloroplasts, demonstrated an accumulation of precursor proteins in the cytosol and an increase in *Hsc70-4* (an Hsp70 isoform) and *CHIP* expression [109]. The Hsc70-4 protein plays a critical role in interacting with the transit peptides of target substrates [109]. Thenceforth, a complex formation with CHIP and substrate bound Hsp70 or 90 will lead to an interaction with an E2 to facilitate the ubiquitination and degradation of target substrates via the 26S proteasome (Figure 1D) [107,109,113].

FtsH and Clp proteases consist of multi-subunit complexes, whereby any alteration to the production of these subunits may result in a disruption of the function and structure of the protease [107,110,114]. AtCHIP can indirectly regulate chloroplast proteases Clp and FtsH by interacting with their cytosolic precursors, including FtsH1, FtsH2, ClpP3, ClpP4, and ClpP5 [107,110,111,113]. For instance, *AtCHIP* overexpression analyses demonstrated a reduction in subunit precursor steady-state levels under high-intensity light conditions [107,111]. Importantly, the overexpression of *AtCHIP* was also observed to prevent the chlorotic phenotypes present in the suppression or overexpression of ClpP4 by restoring proper subunit stoichiometry. These findings have been detected in transgenic tobacco plants as well, suggesting that CHIP plays a conserved role in maintaining chloroplast protease homeostasis and establishing functional protease core complexes [96,107,111].

Recently, CHIP was reported to be strongly affected by ABA and play a crucial role in stress and heat tolerance in Arabidopsis and tomato plants [115,116]. Notably, increased temperature and heat stress has been attributed to a rise in misfolded chloroplast precursor proteins which can also result in decreased protein import into chloroplasts [96,117]. In the absence of CHIP, increased temperature sensitivity, reduced photosynthetic activity, and elevated levels of photosynthetic protein aggregates were observed [96,116]. Interestingly, these aggregates were still ubiquitinated, implying the existence of other unknown E3 ligases involved in chloroplast function [116]. Strikingly, CHIP is also able to interact with two nuclear-encoded proteins important in chloroplast function. This includes the ribulose-biphosphate carboxylases (Rubisco) small subunit (RbcS) and light harvesting complex photosystem II subunit 6 (Lhcb6), which imply an additional role of CHIP in maintaining the chloroplast proteome [118]. Taken together, these findings suggest the need for a rapid response in preventing the accumulation of unimported chloroplast precursor proteins and balancing protease activity under stressful conditions [96,116].

### 2.5. TT3.1, a Plasma Membrane-Localized RING-Type E3 Ligase

Plants are constantly exposed to changing temperatures [19,119,120]. High temperatures may damage many metabolic, photosynthetic, and other various molecular pathways; thus, inhibiting the growth and development of plants as a result of heat stress [119,120,121,122]. For that reason, the ability to sense temperature fluctuations is vital to quickly respond and adapt to the environment [119,123,124]. An important advancement in understanding various resistance mechanisms in plants led to the discovery of Thermo-Tolerance 3.1 (TT3.1). TT3.1 is a plasma membrane-localized RING-type E3 ligase necessary for the ubiquitination and vacuolar degradation of TT3.2, a chloroplast precursor protein and “potential thermosensor” protecting the thylakoids under increased temperatures [125].

In this study, overexpression analysis of TT3.1 or knockdown of TT3.2 both showed a notable increase in grain yield under heat stress [125]. Strikingly, heat stress-induced accumulation of TT3.2 has been shown to cause chloroplast damage, implicating the need for high TT3.1 activity for its rapid degradation. TT3.1-mediated degradation of TT3.2 observed under heat treatment and in transient expression and immunogold-labelling assays revealed that TT3.1 will translocate to the endosomes to intercept and target chloroplast-destined TT3.2 proteins to the endosomes for degradation via vacuoles (Figure 1E). Additionally, both TT3.1 and TT3.2 have been detected in valuable crops, such as rice, maize, and wheat [125]. Thus, suggesting a likely conserved mechanism of thermosensing and highlighting new possible strategies in engineering heat tolerant plants. Despite these findings, the mechanisms underlying plasma membrane-to-chloroplast communication to initiate the process of thermotolerance and how the accumulation of TT3.2 causes damage remains unknown [125].

## 3. Intra-Chloroplast Protein Degradation, the Role of CDC48

CDC48 is a highly conserved eukaryotic protein and ubiquitin-dependent segregase belonging to the AAA+ family [7,39]. Located in the cytosol and nucleus, CDC48 plays an important role in the CHLORAD pathway. Degradation via CHLORAD is achieved by the coordinated actions of SP1, which directly ubiquitinates target substrates, and retrotranslocation of the target substrates to the cytosol by SP2 and CDC48 proteins for degradation via the 26S proteasome (Figure 1F). More specifically, CDC48 provides the proper ATP-powered machinery needed to surpass the physical barriers presented by the chloroplast envelope membranes upon protein extraction [7]. To function, CDC48 requires a complex formation with Ubiquitin Fusion Degradation1 (UFD1)-Nuclear Protein Localization4 (NPL4), a heterodimeric cofactor that binds ubiquitin and small ubiquitin-like modifier proteins [39].

Prior investigation of the CHLORAD pathway suggested that its ubiquitination targets are those of the TOC apparatus [7]. Beyond chloroplast protein import machinery, extensive ubiquitination was recently found in chloroplast fractions, and in greater abundance under UPS or retrotranslocation inhibition. Among these ubiquitinated proteins were the Rubisco large subunit (RbcL) and ATP synthase subunit beta (AtpB), both of which are chloroplast encoded and degraded through the CHLORAD pathway [39]. Most recently, an unpublished study by Sun et al. demonstrated additional targets of the CHLORAD pathway involved in photosynthesis (i.e., electron transport, energy transduction, and carbon fixation), gene expression, and fatty acid/lipid metabolism [126]. One important target substrate of interest is fatty acid export 1 (FAX1), an inner chloroplast envelope protein and key player in fatty acid (FA) export. In the presence of defective CHLORAD-mediated degradation of FAX1, disturbances in cellular metabolic homeostasis (i.e., a notable decrease in cellular lipid species and chloroplast-produced FAs) were observed [126]. Altogether, these findings raise questions about how intra-chloroplast proteins are selected by the CHLORAD pathway and if intra-chloroplast proteins are marked by ubiquitin inside the organelle [39,127]. The presence of internal chloroplast ubiquitination processes and a better understanding of the precise functions of the CDC48 complex under a myriad of stresses await further investigation.

## 4. Outlook

Within the last decade, the discovery of several connections between the UPS and chloroplasts has largely altered our current views about the extent to which E3 ubiquitin ligases can maintain their regulatory activities in plants. The study of E3s in chloroplast function, which has led to the identification of ubiquitinated chloroplast proteins, is valuable for future agricultural applications. For instance, E3 ligases such as TT3.1 can potentially be exploited and re-engineered to control the degradation of TT3.2 for chloroplast survival under high temperatures [125]. Uncovering the diverse mechanisms underlying the interactions of E3 ligases with chloroplast associated proteins will be significant to unveiling deeper insights into how the chloroplast proteome can be manipulated to promote adaptation in the ever-changing environments.

## Data Availability

Not applicable.
